# Corruption in Health Systems: The Conversation Has Started, Now Time to Continue it: Comment on "We Need to Talk About Corruption in Health Systems"

**DOI:** 10.15171/ijhpm.2019.104

**Published:** 2019-11-11

**Authors:** Hongsheng S. Lu, Bing X. Ho, J. Jaime Miranda

**Affiliations:** ^1^Global Health Research Center, Duke Kunshan University, Kunshan, Jiangsu, China.; ^2^Trinity College for Arts & Sciences, Duke University, Durham, NC, USA.; ^3^CRONICAS Center of Excellence in Chronic Diseases, Universidad Peruana Cayetano Heredia, Lima, Peru.; ^4^School of Medicine, Universidad Peruana Cayetano Heredia, Lima, Peru.

**Keywords:** Corruption, Health Systems, Global Health, Peru

## Abstract

Holistic and multi-disciplinary responses should be prioritized given the depth and breadth through which corruption in the healthcare sector can cover. Here, taking the Peruvian context as an example, we will reflect on the issue of corruption in health systems, including corruption with roots within and outside the health sector, and ongoing efforts to combat it. Our reflection of why corruption in health systems in settings with individual and systemic corruption should be an issue that is taken more seriously in Peru and beyond aligns with broader global health goals of improving health worldwide. Addressing corruption also serves as a pragmatic approach to health system strengthening and weakens a barrier to achieving universal health coverage and Sustainable Development Goals related to health and justice. Moreover, we will argue that by pushing towards a practice of normalizing the conversation about corruption in health has additional benefits, including expanding the problematization to a wider audience and therefore engaging with communities. For young researchers and global health professionals with interests in improving health systems in the early career stages, corruption in health systems is an issue that could move to the forefront of the list of global health challenges. This is a challenge that is uniquely multi-disciplinary, spanning the health, economy, and legal sectors, with wider societal implications.

## Tracing Corruption to its Source, Towards a Horizontal Rather Than Vertical Enforcement of Health Systems Integrity

Addressing corruption at the health systems level requires the support of “champions,” individuals committed to reducing corruption and in key positions to influence, and the identification of the foundations of health systems corruption. Due to the short duration of political terms, Dr. Patricia Garcia, former Minister of Health of Peru, believes it’s “hard to try to do things in such a short time [due to political turnover], [so] it’s important to build roots (the beginnings of policy change) for whatever will come next.”^1^ During her tenure as Minister of Health and in her work today, Dr. Garcia has championed the cause of reducing corruption in the Peruvian health system. Concerns that corruption studies could potentially divert attention away from more important issues could be mitigated by political champions who could guide policy formulation and implementation — no matter how big or small — with integrity. Initially, champions are needed to start with, but champions alone will not suffice, and programs raising consciousness about institutional corruption would serve to improve health professionals’ ability to see changes and improvements. In addition, those programs can serve to monitor and refine institutional changes by being able to better document the impact of corruption on current health systems, and the responses provided to it. Ultimately, these changes affect the health and well-being of patients and the entire healthcare workforce.

Careful consideration of the institutional sources and mechanisms that drive and sustain health corruption is crucial. Tracing issues to the source of their corruption would greatly improve the sustainability of anti-corruption efforts. For example, such a profound topic becomes obvious when scrutinizing the perceived normality associated with absenteeism in low- and middle-income country settings.^2-4^ Health workers’ low wages and irregular payments, paired with weak oversight by governance systems, may bolster health workers’ decisions to engage in dual practice and missing work days.

Corruption is usually seen as stemming from informal behaviors that ignores public rules, regulations, and laws.^3^ The problem is that perception of informality shadows and obscure the real penetration of such practices, usually much more common and larger than expected. As such, collective enforcement of health sector honesty could be done by looking to regulatory measures like ensuring that any user, patients and/or providers are aware of mechanisms in place to oversee corruption and are comfortable in using them.

## Strengthening Communication to Support Corruption Research: InsightsFromPeru

In Peru, a government-wide national plan to fight corruption was proposed in 2008,^5^ and in 2012 a High-Level Anticorruption Commission was established.^6^ In 2015, the Ministry of Health adopted its own plan for the health sector.^7^ Yet, exposing corruption can be systematically difficult and dangerous to one’s credibility and career and the challenge comes in the form of reporting and ensuring protection for those who are faced with the pressure to be silenced from higher authority.

Following a Legislative Decree in 2017^8^ designed to empower and protect those who report acts of corruption as well as to punish those who make such reports in bad faith, the Peruvian Ministry of Health launched OTRANS (Office of Transparency and Anticorruption).^9^ A key component of OTRANS was the opening of a new, simplified method for the reporting of corruption through the Internet, by phone, or in writing.^10,11^ The reporting form can be submitted online or physically, and can be made from specific entities within the Ministry of Health and its affiliated hospitals, as well as regional entities. The form asks for a description of the purported corruption, the individual involved, and evidence, if available. The new reporting tool dually serves to increase awareness of corruption and improve the ability to study corruption. A centralized repository for complaints and concrete reports from victims/observers of corruption will reduce officials’ ability to shift the blame of corruption to those with less influence.^10,12^ The establishment of OTRANS and the updated reporting system are improved resources to combat corruption but do not guarantee a health system with less corruption. Additionally, concerns should be raised about the lack of transparency of the investigation and follow up process following the submission of a complaint. Wariness about the integrity of OTRANS is valuable too, and scrutiny can help to ensure that the office is not being manipulated for the ends of management or others.

In addition to becoming a hub for reporting acts of corruption, OTRANS also plays a role in the investigation of corruption. Recently, a group of doctors from a large national public hospital was discovered to be working in private offices during paid working hours.^13,14^ They were also found to offer their private patients unauthorized access to hospital equipment for their medical exams.^13,14^ These practices are not unique to Peru, as absenteeism and diversion of public resources to private operations has also been reported in other settings.^3^ Other forms of corruption, such as bribes to expedite treatment, diversion of patients and irregular equipment and drug procurement, are found in multiple countries and is common knowledge among individuals in the health systems.

As corruption frequently hides behind the red tape of bureaucracy, a simplification of the reporting process is a step in the right direction to improve the exposure and correct the internal issues of corruption. In the same vein, publicly exposing and discussing the types, location, and further investigation of the corruption complaints received by the OTRANS office is needed to normalize the conversation about corruption. In so doing, this practice of public accountability will ensure that the ingrained view of “corruption happens and does not bother” moves away from being the norm.

## Corruption Outside the Health Sector Also Affects the Health System

Solutions to finding common ground over the creation and institutionalization of new health systems protected against corruption should strive to protect workers and patients while giving them a larger voice in the process of changing the current system.^3,7^ The yield of these mechanisms in overturning current practices may be minimal if the root of the problems are wider societal crisis. One-size-fits-all type-of-recommendations will not necessarily solve major fundamental weaknesses of the health system when contexts for individual corrupt practices vary.

Whilst addressing corruption within healthcare delivery practices appears as the most immediate angle of corruption within health systems, there are other macro-level areas where corruption occurs. Corruption in this form operates at higher levels in the system, at state level and beyond, and as a result tend to have broader reach and scale.^6,15,16^Corruption in equipment purchasing, construction, and the pharmaceutical industry, among others, impact the effectiveness of health systems and is often centered around competing interests, sometimes within legal frameworks.

When private interests are at odds with the greater good and public interest, the public interest can be put to the side as a result of state capture from suppliers looking to turn a profit for their investors.^15^ Laws and policies compromised to benefit private economic gain is not only the result of corruption but fosters continued and increased corruption. An example that has received some attention is within the pharmaceutical industry. New drugs, when first introduced to the market, can be monopolized for increased profit by the drug companies that produce them. In order to maximize the window of time that drugs can be sold a monopoly prices, companies accelerate and expedite the clinical trials process to push the drugs to market as soon as possible.^17^ Among a number of morally grey and ethically ambiguous behaviors employed to achieve this goal, researchers hired by companies will lobby policy-makers and influence laws to their benefit, at the expense of data about drug safety and the rights of those involved in trials. The pharmaceutical industry has also been found to mobilize patient groups.^18^ Beyond lobbying, companies also install ministers and other key figures friendly to their interests, directly allowing influence over laws responsible for the regulation of these companies.

In addition to the concept of capturing state regulators, broad political corruption also directly affects health systems and their effectiveness. Although there is some overlap between the concepts of political corruption and state capture, political corruption is more associated with exchanges between high levels of government and private industry for advantages, often related to investment and procurement.^19^ Corruption in the construction industry illustrates this well, as described by Kenny: “Construction firms bribe in order to obtain contracts, to increase profit margins on those contracts and to reduce the cost of construction.”^20^ As a result of corruption in construction, not only are health standards compromised but also increased costs of maintenance may be placed on health systems as a result of poor infrastructure. Also, political corruption directly affects health system in the area of procurement.

A critical analysis of how the private sector infiltrates not just the health system but also the regulatory and legal infrastructure around the health system needs to be undertaken to better understand and combat corruption. An extension of this is the transition of senior officials between the public and private sector, facilitating certain arrangements when in positions of influencing decision making in the public sector, and then transitioning to the private sector.^21^ These external influences of corruption have a broad reach beyond just the health sector, and has been receiving increasing attention in some parts of the world. Just as progress is being made to reduce corruption in health sectors, attention should be paid to these external sources of corruption as well, where financial incentives from the private sector plays a large role.

## Advancing Towards Corruption Research

Furthermore, the mechanisms of corruption research can only be as good as the infrastructure protecting those who choose to undertake such investigations. Consequently, the nature of systematic efforts to reduce health system corruption could be learned from existing reports on potential steps to protect the independence and integrity of global health research.^22^

Corruption research, like any other type of research, will likely be funded by major donors, as this corruption in healthcare can well be a litmus test for implementation science.^23^ Independent committees, consisting of key stakeholders and representatives from the community under study, could be established to impede commissioning bodies’ interference with the collection, analysis, and dissemination of data. Forcing commission agencies to clearly state their intent behind researching a health system should reduce pressure on researchers to produce favorable results and protect the body under investigation from wrongful or misunderstanding censure.

Guaranteeing transparency and accountability are essential requisites to advance towards minimizing corruption in health systems. The independent offices and institutions responsible for conducting corruption investigations could safeguard themselves by encouraging senior faculty in academia to lead discussions between funders and implementers to deny unjust contractual provisions and encourage the publication of positive and negative research findings. Outlining a process for releasing controversial and politically charged results by senior staff would set a standard for junior investigators to work towards corruption-free research, and ultimately a corruption-free working environment, as learned on research integrity from other fields.^22^ This creates two perspectives to consider on how to best conduct research for the improvement of medical service delivery in the long run: ensuring corruption research does not harm the current health systems’ ability to treat patients, but also pushing for greater transparency in the interest of patient safety and risk exposure.^18^

## The Future: Towards a Corruption-Free Environment

The efforts in Peru may not be isolated and experiences around the globe aimed towards addressing corruption in health systems may already be in place, albeit at different stages. Corruption in some forms has become normalized, because of how it sometimes is understood as a means to allow an otherwise dysfunctional health system to operate. Often, corruption is a well-known and acknowledged part of a health system.^24^ Just because it is the norm, however, does not mean that it is acceptable. Corruption that has become a part of the system should be re-problematized and pushed back into the discussion of health systems challenges.

Equally important, being upfront about corruption should fall to those in training and future leaders—corruption should be better integrated into curricula for both researchers and healthcare providers and awareness should be increased. For young researchers and global health professionals in the early career stages, corruption in health systems is an issue that could move to the forefront of the list of global health challenges. This is a challenge that is uniquely multidisciplinary, spanning the health, economy, and legal sectors, with wider societal implications, much in line with the multidisciplinarity required to advance global health.^25,26^

Discussion of corruption in health systems can affect discourse beyond academia and the medical field to politics, an essential player in facing corruption.^27^ Transparency International’s definition of corruption as “the abuse of entrusted power for private gain”^28^ is a general but foundational point in the discussion. As a primarily underexposed issue, the idea of corruption tends to narrowly frame the concept around individual corruption. Other forms of corruption that fall less neatly into this definition are less acknowledged and undermine health systems without much notice from academics or the rest of the world beyond large scale scandals. This theme rings true in global health education as well, despite documented evidence that corruption in health systems costs lives and money.^29,30^ By teaching about the challenges of corruption in health systems early on in the careers of the next generation of leaders, awareness will be improved, and, more importantly, the discussion on corruption will be continued.

In pursuing this goal, how do we define corruption, especially when introducing it as a topic to students who may not yet have had exposure or practice in settings where they could have witnessed it firsthand? Consciousness of corruption, risks related to engaging corruption, and transparency are related topics with subtleties that need to be well understood. How can students engage in research on corruption that wholly captures situations instead of focusing on a single facet or misdirecting efforts to scapegoats? The evidence on the effectiveness of interventions to target different types of corruption is scant.^12^ It will fall on the shoulders of the next generation of researchers and leaders to sustain the conversation around corruption, build a body of evidence on effective interventions and develop innovative responses, and work towards the goal of corruption-free healthcare systems.

## Final Remarks

A number of obstacles still stand in the way of approaching corruption as a health challenge. Among the many solutions and proposals to deal with corruption, a common thread of continued, sustained discourse can be identified. From identifying champions to better address corruption and establishing oversight to monitor both corruption and corruption research, an increase in the public and academic eye on corruption in health systems is key to problematizing the issue and making the reduction (or eradication!) of corruption a priority. A longer term approach to achieving this goal is through leveraging on opportunistic environments provided by the years of education and training of future healthcare professionals. Generally speaking, how can we teach and study corruption in a way that supports the goals of global health, and how can we learn and develop new ways to combat corruption? Questions still remain in the effort to take a stand against corruption, but it is clear that continued, sustained dialogue will be essential to accomplish this task, mainly because corruption that affect health and well-being occurs within and outside the health sector. Actions conducting towards a corruption-free environment need to be taken but close consideration must also be given to the importance and feasibility of those actions. And, in particular, those actions need to become the norm rather than the exception as we push away from a society with systemic corruption and work towards the goal of corruption-free healthcare systems.

## Acknowledgements

We would like to express our gratitude to Dr. Camila Gianella, PhD and to Dr. Gonzalo Gianella, MD for providing critical inputs to earlier versions of this article.

HSL acknowledges having received support from the Duke Kunshan University Global Health graduate studies fund and the Duke University Dean’s Research Award for Master’s Students. BXH acknowledges having received support from the Kenan Institute for Ethics Duke Engage grant at Duke University. JJM acknowledges having received support from the Alliance for Health Policy and Systems Research (HQHSR1206660), the Bernard Lown Scholars in Cardiovascular Health Program at Harvard T.H. Chan School of Public Health (BLSCHP-1902), Bloomberg Philanthropies, FONDECYT via CIENCIACTIVA/CONCYTEC, British Council, British Embassy and the Newton-Paulet Fund (223-2018, 224-2018), DFID/MRC/Wellcome Global Health Trials (MR/M007405/1), Fogarty International Center (R21TW009982, D71TW010877), Grand Challenges Canada (0335-04), International Development Research Center Canada (IDRC 106887, 108167), Inter-American Institute for Global Change Research (IAI CRN3036), Medical Research Council (MR/P008984/1, MR/P024408/1, MR/P02386X/1), National Cancer Institute (1P20CA217231), National Heart, Lung and Blood Institute (HHSN268200900033C, 5U01HL114180, 1UM1HL134590), National Institute of Mental Health (1U19MH098780), Swiss National Science Foundation (40P740-160366), Wellcome (074833/Z/04/Z, 093541/Z/10/Z, 107435/Z/15/Z, 103994/Z/14/Z, 205177/Z/16/Z, 214185/Z/18/Z) and the World Diabetes Foundation (WDF15-1224).

## Ethical issues

Not applicable.

## Competing interests

Authors declare that they have no competing interests.

## Authors’ contributions

JJM conceived the idea together with HSL and BXH. HSL and BXH did the literature search and drafted the first version of the manuscript with substantial inputs of the final version from JJM. JJM revised and edited the manuscript. All authors reviewed and approved the final version.

## Authors’ affiliations

^1^Global Health Research Center, Duke Kunshan University, Kunshan, Jiangsu, China. ^2^Trinity College for Arts & Sciences, Duke University, Durham, NC, USA. ^3^CRONICAS Center of Excellence in Chronic Diseases, Universidad Peruana Cayetano Heredia, Lima, Peru. ^4^School of Medicine, Universidad Peruana Cayetano Heredia, Lima, Peru.

## References

[1] Kaplan J. Translating Research for Better Policy: Advice from Dr. Patricia García, Former Minister of Health of Peru. Medium. September 20, 2018. https://medium.com/@VoicesHSPH/translating-research-for-better-policy-advice-from-dr-cf53df09f82b. Accessed August 10, 2019.

[2] Jumpa M, Jan S, Mills A (2007). The role of regulation in influencing income-generating activities among public sector doctors in Peru. Hum Resour Health.

[3] Onwujekwe O, Agwu P, Orjiakor C (2019). Corruption in Anglophone West Africa health systems: a systematic review of its different variants and the factors that sustain them. Health Policy Plan.

[4] McPake B, Russo G, Hipgrave D, Hort K, Campbell J (2016). Implications of dual practice for universal health coverage. Bull World Health Organ.

[5] Plan Nacional de Lucha contra la Corrupción. Un compromiso de todos. Presidencia del Consejo de Ministros; 2008. http://www.pcm.gob.pe/InformacionGral/plan_anticorrupcion/plan_anticorrupcion.pdf. Accessed August 20, 2019.

[6] Lucha frontal contra la corrupción: Una política diferente para un país distinto. Presidencia del Consejo de Ministros. http://www.pcm.gob.pe/lucha-frontal-contra-la-corrupcion-una-politica-diferente-para-un-pais-distinto/. Accessed August 20, 2019.

[7] Dirección General de la Defensoría de la Salud, Ministerio de Salud. Plan de Lucha Contra la Corrupción en el Ministerio de Salud 2015-2016. Lima: Ministerio de Salud; 2015. http://bvs.minsa.gob.pe/local/MINSA/3253.pdf.

[8] Decreto Legislativo N° 1327. Decreto Legislativo que establece medidas de protección para el denunciante de actos de corrupción y sanciona las denuncias realizadas de mala fe. El Peruano; 2019. http://busquedas.elperuano.pe/normaslegales/decreto-legislativo-que-establece-medidas-de-proteccion-para-decreto-legislativo-n-1327-1471010-6/. Accessed August 20, 2019.

[9] OTRANS: Oficina de Transparencia y Anticorrupción. Ministerio de Salud; 2019. https://www.minsa.gob.pe/otrans/?op=81&tall=7. Accessed August 20, 2019.

[10] Denuncias Anticorrupción. Ministerio de Salud; 2019. https://www.minsa.gob.pe/otrans/denuncias/. Accessed August 20, 2019.

[11] Ministerio de Salud lanza campaña para combatir actos de corrupción. RPP; 2016. https://rpp.pe/lima/actualidad/ministerio-de-salud-lanza-campana-para-combatir-actos-de-corrupcion-noticia-1001588. Accessed August 20, 2019.

[12] Gaitonde R, Oxman AD, Okebukola PO, Rada G (2016). Interventions to reduce corruption in the health sector. Cochrane Database Syst Revm.

[13] Hospital Loayza: médicos atienden en clínicas privadas en sus horas pagadas por Minsa. América Noticias; 2019. https://www.americatv.com.pe/noticias/actualidad/doctores-hospital-loayza-pacientes-medicos-n352219. Accessed August 20, 2019.

[14] Médicos del Loayza atienden en clínicas privadas en horas de trabajo para el Estado. Diario Correo; 2018. https://diariocorreo.pe/edicion/lima/medicos-del-loayza-atienden-en-clinicas-privadas-en-horas-de-trabajo-para-el-estado-video-860808/. Accessed August 20, 2019.

[15] Hussmann K. Addressing corruption in the health sector: securing equitable access to health care for everyone. U4 Issue. 2011:1. https://www.cmi.no/publications/file/3934-addressing-corruption-in-the-health-sector.pdf. Accessed August 20, 2019.

[16] Watts J. Operation Car Wash: The biggest corruption scandal ever? The Guardian; 2017. http://www.theguardian.com/world/2017/jun/01/brazil-operation-car-wash-is-this-the-biggest-corruption-scandal-in-history. Accessed August 20, 2019.

[17] Ugalde A, Homedes N (2015). [The impact of researchers loyal to Big Pharma on the ethics and quality of clinical trials in Latin America]. Salud Colect.

[18] Sample I. Big pharma mobilising patients in battle over drugs trials data. The Guardian. July 21, 2013. http://www.theguardian.com/business/2013/jul/21/big-pharma-secret-drugs-trials. Accessed August 20, 2019.

[19] The rationale for fighting corruption. CleanGovBiz Initiative, OCED; 2014. https://www.oecd.org/cleangovbiz/49693613.pdf. Accessed August 20, 2019.

[20] Kenny C. Construction, corruption, and developing countries. Policy, Research working paper No. WPS 4271. Washington, DC: World Bank; 2007.

[21] Puertas que giran sin parar. Salud con lupa. https://saludconlupa.com/reportajes/la-salud-en-la-mesa-del-poder/puertas-que-giran-sin-parar/. Accessed August 20, 2019.

[22] Storeng KT, Abimbola S, Balabanova D (2019). Action to protect the independence and integrity of global health research. BMJ Glob Health.

[23] Geng EH, Peiris D, Kruk ME (2017). Implementation science: Relevance in the real world without sacrificing rigor. PLoS Med.

[24] Hutchinson E, Balabanova D, McKee M (2019). We need to talk about corruption in health systems. Int J Health Policy Manag.

[25] Rowson M, Willott C, Hughes R (2012). Conceptualising global health: theoretical issues and their relevance for teaching. Global Health.

[26] Rowson M, Smith A, Hughes R (2012). The evolution of global health teaching in undergraduate medical curricula. Global Health.

[27] Mackey TK, Kohler JC, Savedoff WD (2016). The disease of corruption: views on how to fight corruption to advance 21(st) century global health goals. BMC Med.

[28] Transparency International. What is Corruption? Transparency International; 2019. https://www.transparency.org/what-is-corruption. Accessed August 20, 2019.

[29] Hanf M, Van-Melle A, Fraisse F, Roger A, Carme B, Nacher M (2011). Corruption kills: estimating the global impact of corruption on children deaths. PLoS One.

[30] Dreher A, Herzfeld T. The economic costs of corruption: a survey and new evidence. SSRN; 2005. 10.2139/ssrn.734184.

